# Acute Abdominal Pain in Pregnancy Revealing a Wandering Spleen With Infarction: A Case Report

**DOI:** 10.1155/cris/6307093

**Published:** 2026-01-08

**Authors:** Youssef T. Youssef, Mohamed Baklola, Ahmed Elshazli, Naji Al-bawah, Mohamed Abdelhai Mahmoud, Tamer Youssef

**Affiliations:** ^1^ Internship Program, Faculty of Medicine, Mansoura University, 60El-Gomhoria Street, Mansoura, 35516, Egypt, mans.edu.eg; ^2^ General and Endocrine Surgery, Mansoura University Hospitals, Mansoura, Egypt, mans.edu.eg; ^3^ Internship Program, Faculty of Medicine, Sana’a university, Sana’a, Yemen, su.edu.ye

**Keywords:** acute abdomen, pregnancy-induced infarction, spleen infarction, wandering spleen

## Abstract

**Introduction and Importance:**

Wandering spleen (WS) is a rare condition caused by the absence or laxity of splenic suspensory ligaments, predisposing the spleen to displacement and potential complications. While torsion is the most commonly reported issue, infarction due to vascular compromise is a serious and rare complication, particularly in pregnancy. We present a case of splenic infarction in a WS during pregnancy, emphasizing diagnostic challenges and management strategies.

**Case Presentation:**

A 19‐year‐old primigravida at 5 months of gestation presented with progressively worsening abdominal pain and a palpable right‐sided abdominal mass. Ultrasound and magnetic resonance imaging (MRI), chosen for its safety in pregnancy, confirmed an enlarged, ectopic spleen with infarction and splenic vein thrombosis. Conservative management with anticoagulation and supportive therapy was initially attempted but failed due to worsening pain and clinical deterioration. Surgical intervention was deemed necessary, and a splenectomy was performed. The patient recovered well postoperatively, with fetal well‐being maintained throughout.

**Clinical Discussion:**

Diagnosing WS in pregnancy is challenging due to nonspecific symptoms and limited imaging options. MRI plays a pivotal role in identifying ectopic spleen and assessing vascular compromise. Although torsion is commonly associated with infarction, infarction can occur through other mechanisms such as venous thrombosis or outflow obstruction. Early recognition and timely surgical intervention are essential to reduce maternal and fetal morbidity.

**Conclusion:**

WS in pregnancy presents a diagnostic and therapeutic challenge, with infarction posing a significant but underreported risk. MRI is a valuable tool in pregnant patients, allowing safe and accurate diagnosis. A multidisciplinary approach and individualized treatment strategies are essential for optimizing both maternal and fetal outcomes.

## 1. Introduction

The spleen is normally fixed in the left upper quadrant of the abdomen by peritoneal ligaments, including the gastrosplenic and splenorenal ligaments. However, in rare cases, these ligaments may be underdeveloped or lax, leading to a condition known as wandering spleen (WS) [1]. The incidence of WS is less than 0.2% in the general population, with a higher prevalence among women of reproductive age, likely due to hormonal influences on ligamentous laxity during pregnancy [2]. However, the true incidence of WS specifically during pregnancy remains unknown because most cases are reported as isolated case reports or small series.

Clinical manifestations range from asymptomatic abdominal mobility to acute abdominal pain due to complications such as torsion, infarction, or splenic vein thrombosis [3, 4]. These presentations often mimic other obstetric or surgical emergencies, which may delay diagnosis. Imaging plays a crucial diagnostic role. While Doppler ultrasound is typically the first‐line tool for assessing splenic position and vascularity [5], magnetic resonance imaging (MRI) is preferred during pregnancy due to its superior soft‐tissue contrast and lack of ionizing radiation [6, 7].

Management depends on symptom severity and splenic viability. Conservative therapy, including anticoagulation, may be appropriate in stable cases with thrombosis, but splenopexy or splenectomy is indicated in complicated cases such as infarction or rupture [8]. Pregnancy adds diagnostic and therapeutic challenges due to anatomical changes, increased intra‐abdominal pressure, and concerns regarding fetal safety [9].

We present a rare case of WS with complete splenic infarction and splenic vein thrombosis in a 19‐year‐old pregnant woman, highlighting the diagnostic utility of MRI and the importance of a multidisciplinary approach for optimal maternal and fetal outcomes.

## 2. Case Presentation

A 19‐year‐old primigravida at 20 weeks of gestation presented to the emergency department with progressively worsening abdominal pain over 2 weeks. The pain was localized to the right lumbar and periumbilical regions and was associated with a palpable abdominal mass. She had no previous history of abdominal surgery, trauma, or chronic illness, and her bowel habits were normal.

On examination, the patient appeared distressed but was hemodynamically stable. Abdominal examination revealed a gravid uterus corresponding to 20 weeks of gestation, with the uterine fundus palpable just below the umbilicus. A separate, firm, mobile, and tender mass measuring approximtely 12 by 10 cm was felt in the right paraumbilical and lumbar regions, distinct from the uterus, and there were no signs of peritoneal irritation.

Pelviabdominal ultrasound demonstrated an ectopic spleen in the right paraumbilical region with moderate splenomegaly. Grayscale and Doppler imaging revealed heterogeneous parenchymal echotexture and avascular hypoechoic areas suggestive of splenic infarction, along with a dilated, thrombosed splenic vein at the hilum (Figure 1). The uterus contained a single live intrauterine fetus with normal cardiac activity and biometric measurements corresponding to gestational age.

Figure 1Ultrasound findings of an ectopic spleen with splenic infarction in a pregnant patient. A series of grayscale and Doppler ultrasound images demonstrating an ectopic and malrotated spleen located in the supra‐umbilical and right paraumbilical region. (A,B) The abnormal splenic position, distorted vascular hilum, and mild splenic enlargement (labeled by white line). (C,D) In homogeneous parenchymal echotexture with peripheral hypoechoic, avascular areas consistent with splenic infarction (labeled by yellow arrow).

**Figure:**
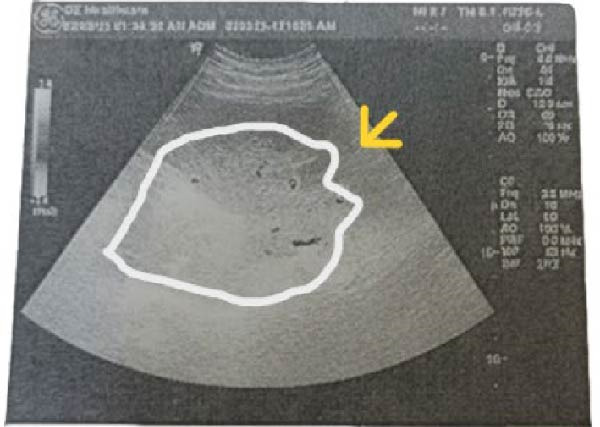
(A)

**Figure:**
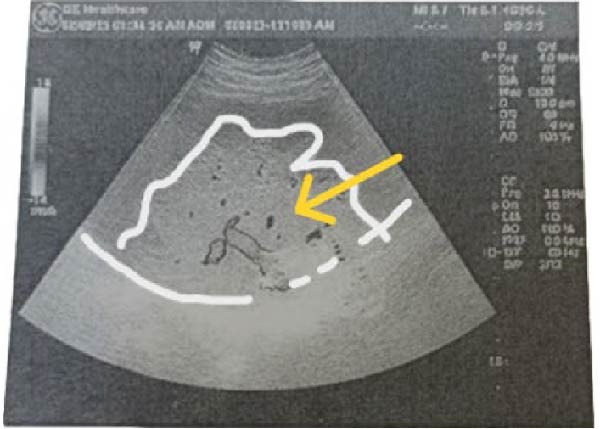
(B)

**Figure:**
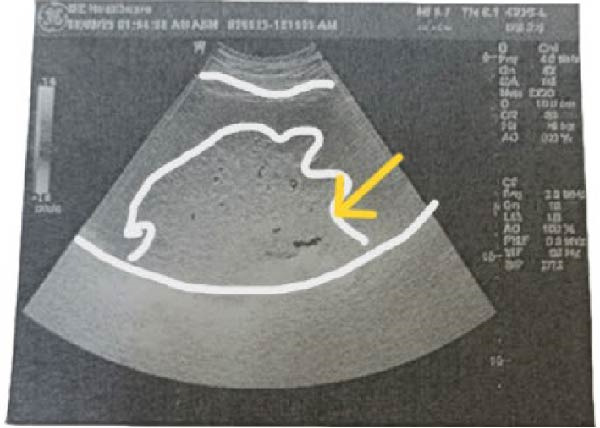
(C)

**Figure:**
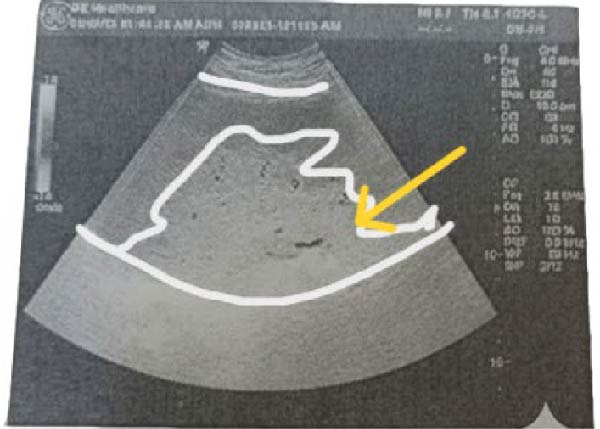
(D)

**Figure 2:**
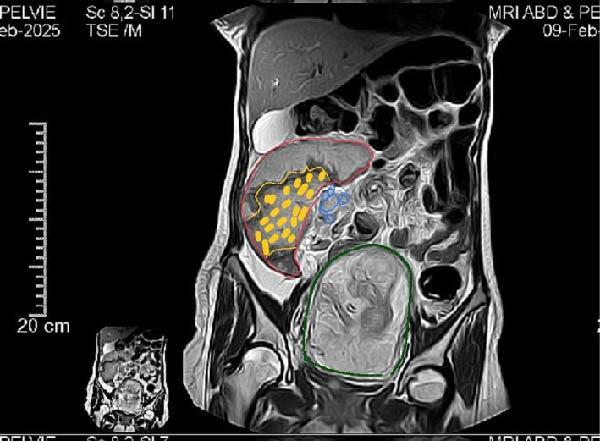
MRI findings of wandering spleen with infarction and splenic vein thrombosis, (green) represents gravid uterus, (blue) represents aneurysmal dilatation of splenic vein stagnant flow, (red) represents enlarged wandering spleen, (yellow) represents splenic infarct.

MRI was subsequently performed to avoid ionizing radiation. The scan confirmed the absence of the spleen from its normal anatomical position and revealed a large ectopic spleen in the right lumbar area. The parenchyma appeared heterogeneous with central geographic hypointensity consistent with infarction, and there was evidence of splenic vein thrombosis (Figure 2).

Laboratory investigations showed severe anemia with a hemoglobin level of 5.7 g/dL, an elevated total leukocyte count of 14,600 per *μ* L with neutrophil predominance, a C‐reactive protein level of 82 mg/L, and an increased lactate dehydrogenase level of 618 U/L. Liver function tests were within normal limits (AST 24 U/L, ALT 20 U/L, total bilirubin 0.7 mg/dL). The patient received a blood transfusion and was admitted for conservative management consisting of intravenous broad‐spectrum antibiotics, analgesics, bowel rest with nasogastric decompression, and therapeutic anticoagulation with enoxaparin (Clexane) 80 mg once daily.

Conservative therapy was continued for 5 days; however, the patient’s pain progressively worsened, raising concern for complete splenic infarction or hemorrhagic complications. The clinical diagnosis at this stage was WS with splenic infarction and splenic vein thrombosis in pregnancy.

A multidisciplinary team involving obstetrics, anesthesia, intensive care, and general surgery specialists decided to proceed with surgical intervention due to clinical deterioration. Progestin support (Prontogest) was administered to maintain pregnancy, and blood products were prepared, including four units of packed red blood cells and four units of fresh frozen plasma. High‐risk informed consent was obtained, and the patient was transferred to the operating room for an urgent exploratory laparotomy.

**Figure 3:**
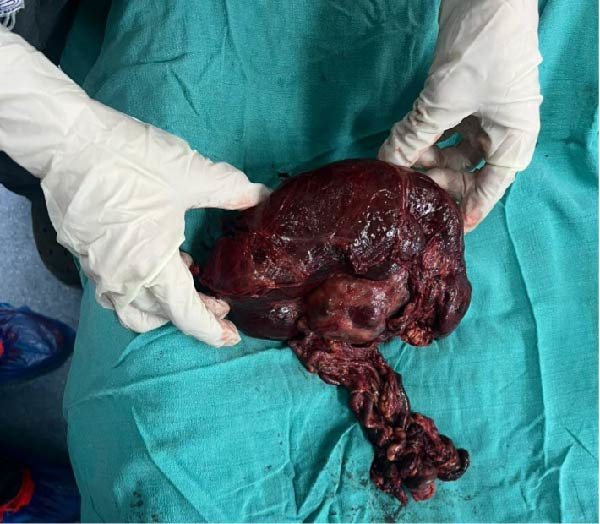
Excised wandering spleen with infarction following splenectomy in a pregnant patient.

Through an upper midline incision, a large omental mass was observed covering the viscera. Dissection revealed a necrotic, enlarged spleen located in the right lumbar region. The splenic vein and hilum were thrombosed with aneurysmal dilation. A splenectomy was performed after ligation of the vascular pedicle, and the infarcted spleen was removed. Gross examination confirmed complete infarction and thrombosis (Figure 3). Two abdominal drains were placed, one in the pelvis and another in Morrison’s pouch, and the abdomen was closed in layers. The patient was transferred to the intensive care unit for close maternal and fetal monitoring. Postoperatively, recovery was uneventful, and fetal viability was maintained throughout the hospital stay.

## 3. Discussion

WS is an uncommon condition that results from congenital or acquired laxity or absence of the splenic suspensory ligaments, allowing the spleen to migrate from its usual position in the left upper quadrant. This abnormal mobility predisposes the organ to complications such as torsion, infarction, or splenomegaly. Although WS may occur at any age, it predominantly affects women of reproductive age, likely due to hormonal effects on connective tissue elasticity during pregnancy [10–13].

Physiological changes during pregnancy, including increased intra‐abdominal pressure and progesterone‐induced ligamentous relaxation, further contribute to splenic displacement [10]. While torsion is the most commonly reported complication of WS in pregnancy, the current case highlights a rare occurrence of complete splenic infarction secondary to splenic vein thrombosis in the absence of torsion. Similar reports in the literature describe torsion‐related infarction rather than pure vascular thrombosis [3, 14]. Zhou et al. [15] reported torsion of a pelvic WS with partial infarction, confirmed radiologically by a twisted splenic pedicle and the classic “whirl” sign. Our case expands the clinical spectrum by demonstrating a non‐torsion‐related infarction mechanism due to venous occlusion.

The clinical presentation of WS is highly variable and often nonspecific. Patients may experience recurrent or acute abdominal pain, nausea, vomiting, or a palpable mobile mass, which may mimic obstetric or gastrointestinal pathologies and lead to diagnostic delays [16, 17]. In this patient, the presence of a distinct, mobile right‐sided abdominal mass separate from the gravid uterus suggested an extrinsic intra‐abdominal pathology rather than uterine origin.

Ultrasound with Doppler is an essential first‐line tool for assessing splenic location and perfusion. In this case, ultrasound revealed an ectopic, avascular spleen, suggesting infarction, which was subsequently confirmed by MRI (Figure 1). MRI is particularly valuable in pregnancy because it provides detailed soft tissue contrast and vascular information without exposing the fetus to ionizing radiation [6]. Although computed tomography is typically the diagnostic standard in nonpregnant patients, it is generally avoided in pregnancy except in life‐threatening situations [18].

Management strategies depend on symptom severity and splenic viability. Asymptomatic or incidentally discovered WS can be managed electively with splenopexy to preserve splenic function [16]. However, once infarction or thrombosis occurs, splenectomy becomes the definitive treatment to prevent further complications such as rupture, hemorrhage, or sepsis [3, 11]. In our case, despite initial conservative management with anticoagulation and supportive measures, the patient’s clinical deterioration necessitated surgical intervention.

Laparoscopic splenopexy or splenectomy has been successfully reported for WS in nonpregnant patients and selected pregnant cases, offering reduced postoperative pain, shorter hospital stay, and faster recovery [19]. However, laparoscopy in pregnancy requires careful consideration of gestational age, uterine size, and maternal positioning to avoid compromised uteroplacental perfusion. Given the patient’s second‐trimester status, marked splenomegaly, and concern for infarction, open laparotomy was the safer and more practical approach in this emergency setting [3, 10–12].

Emergency non‐obstetric surgery during pregnancy carries inherent risks, including preterm labor, fetal loss, and maternal morbidity, which vary by gestational age and the urgency of intervention. The second trimester is generally considered the safest period for necessary surgical procedures, as the risk of teratogenicity is minimal and uterine size allows adequate surgical access [19]. In this case, careful preoperative optimization—including correction of severe anemia, hormonal support for pregnancy maintenance, and perioperative coordination among obstetric, anesthesia, surgical, and intensive care teams—contributed to a favorable outcome.

Postoperatively, the patient recovered without maternal or fetal complications. She received standard postsplenectomy vaccinations against encapsulated organisms such as *Streptococcus pneumoniae*, *Haemophilus influenzae*, and *Neisseria meningitidis*, in accordance with established recommendations [20]. Long‐term follow‐up is crucial to prevent overwhelming postsplenectomy infection (OPSI), a rare but potentially fatal complication [20].

This case underscores the importance of maintaining a high index of suspicion for WS in pregnant patients presenting with unexplained abdominal pain and a palpable mass. MRI plays a pivotal role in diagnosis, while prompt surgical management supported by a multidisciplinary team is essential for optimizing maternal and fetal outcomes.

## 4. Conclusion

WS is a rare but serious condition in pregnancy that can lead to infarction and acute abdomen. MRI offers a safe and accurate diagnostic tool, while timely surgical intervention remains crucial when conservative management fails. Early recognition and a coordinated multidisciplinary approach are essential to achieving favorable maternal and fetal outcomes.

## Consent

Written informed consent was obtained from the patient for the publication of this case report and any accompanying images. All identifying information has been removed to ensure patient anonymity.

## Conflicts of Interest

The authors declare no conflicts of interest.

## Author Contributions

Youssef T. Youssef and Mohamed Baklola contributed equally to the conception, drafting, and final approval of the manuscript. Ahmed Elshazli was responsible for surgical case management and intraoperative documentation. Naji Al‐bawah contributed to critical revision and literature review. Mohamed Abdelhai Mahmoud assisted in radiological diagnosis and data interpretation. Tamer Youssef contributed to patient follow‐up and postoperative assessment. Youssef T. Youssef and Mohamed Baklola contributed equally to this work.

## Funding

No funding was received for this manuscript.

## Data Availability

The data that support the findings of this study are available from the corresponding author upon reasonable request.
